# The plasma proteomic signature as a strategic tool for early diagnosis of acute coronary syndrome

**DOI:** 10.1186/1477-5956-12-43

**Published:** 2014-10-10

**Authors:** Carlos M Laborde, Sergio Alonso-Orgaz, Laura Mourino-Alvarez, José Moreu, Fernando Vivanco, Luis R Padial, María G Barderas

**Affiliations:** Laboratory of Vascular Physiopathology, Hospital Nacional de Parapléjicos, SESCAM, Toledo, Spain; Department of Hemodynamic, Hospital Virgen de la Salud, SESCAM, Toledo, Spain; Department of Immunology, IIS-Fundación Jiménez Diaz, Madrid, Spain; Department of Biochemistry and Molecular Biology I, Universidad Complutense, Madrid, Spain; Department of Cardiology, Hospital Virgen de la Salud, Toledo, Spain

**Keywords:** Acute coronary syndrome, Protein, Biomarker, Two-dimensional fluorescence difference in gel electrophoresis

## Abstract

**Background:**

Acute coronary syndrome is the major cause of death in developed countries. Despite its high prevalence, there is still a strong need for new biomarkers which permit faster and more accurate diagnostics and new therapeutic drugs. The basis for this challenge lay in improving our understanding of the whole atherosclerotic process from atherogenesis to atherothrombosis. In this study, we conducted two different proteomic analyses of peripheral blood plasma from non-ST elevation acute coronary syndrome and ST elevation acute coronary syndrome patients *vs* healthy controls.

**Results:**

Two-dimensional Fluorescence Difference in Gel Electrophoresis and mass spectrometry permitted the identification of 31 proteins with statistical differences (p < 0.05) between experimental groups. Additionally, validation by Western blot and Selected Reaction Monitoring permitted us to confirm the identification of a different and characteristic plasma proteomic signature for NSTEACS and STEACS patients.

**Conclusions:**

We purpose the severity of hypoxia as the cornerstone for explaining the differences observed between both groups.

## Background

Acute coronary syndromes (ACS) remains the leading cause of death in developed countries and presents an increasing prevalence in developing countries [1]. Despite its high prevalence, there is currently no biomarker to detect the degenerative process that causes the ACS before their clinical manifestation to appear.

In the last two decades there have been major advances in understanding the pathophysiology of atherogenesis, especially through a better knowledge of the role that inflammation plays in the different stages of plaque development.

Currently, an increasing importance is assumed to the study of the biology and activity of the atherosclerotic plaque. Both factors are considered more important to determine whether a lesion is going to produce an ACS rather than the degree of luminal stenosis [2]. It has been found that biologically active lesions have several characteristics that distinguish them from the more inactive plaques. These include morphological and inflammatory features, which enhance a large infiltrate of macrophages and lymphocytes, a high production of molecules that destroy the cytoskeleton of the plaque (cytokines, metalloproteinases, etc.) and a decrease in protein synthesis, such as collagen.

It is further known that an adaptive remodelling of the injured artery accompanies the growth of the atherosclerotic plaque. At this stage, the analysis of the coronary lumen does not identify any alteration, while analysis of the arterial wall itself shows its existence. The progressive increase in our knowledge of the intimate relationship between inflammation and atherogenesis has allowed a better understanding of the pathophysiology of this disease [3, 4]. It has additionally led to the appearance of a large number of studies [5–10], which have discovered potential biomarkers and new therapeutic targets to establish a better and earlier diagnosis of the disease and initiate appropriate treatment to improve the prognosis of patient survival.

Current clinical criteria for the diagnosis of ACS established by the European Society of Cardiology (ESC) and the American Heart Association (AHA) include the determination of plasma levels of troponin and the heart type creatine kinase isoenzyme (CKMB) [11]. However, in both cases, their plasma levels are not elevated until 4–6 hours after onset of symptoms, which undoubtedly leads a delay in diagnosis.

In this sense, it is necessary the identification of new biomarkers transferable to clinical practice. Its introduction would help to establish an early diagnosis of the disease, could provide new therapeutic targets on which to develop drugs, or could permit monitoring the evolution of patients on a specific treatment and thus to establish a correct prognosis.

In the present study, the objective was to discover whether there was a common protein profile between patients who have suffered a non-ST segment elevation acute coronary syndrome (NSTEACS) and patients who have suffered a ST segment elevation acute coronary syndrome (STEACS) or whether the greater hypoxia at which the myocardium is subjected in the second case and consequently the greater extent of tissue necrosis produced the existence of a characteristic proteomic profile.

### Why could be hypoxia important in ACS proteome?

Biochemical disorders that occur in ischemic heart disease are consequence of hypoxia. Under restricted oxygen conditions, the muscular cells of the myocardium obtain energy from anaerobic glycolysis, resulting in lactate accumulation. This excess of lactate causes an intracellular acidosis that leads to the activation of proteolytic enzymes, to degradation of structural proteins responsible for cardiac contractility, to the loss of integrity of the cell membrane and, ultimately, to the release of proteins to the blood and to the intercellular gap due to cell death.

In this process, the proteins that first appear in the circulation are cytoplasmic (myoglobin and cytoplasmic creatine kinase), followed by mitochondrial proteins (mitochondrial creatine kinase) and structural proteins (troponins and myosin). This explains why different plasma levels of these current clinical biomarkers permit the clinicians to theoretically classify the extension of an ACS.

## Results

The Differential In-gel Analysis (DIA) module detected 1293 and 1884 protein spots in the Two-dimensional Fluorescence Difference in Gel Electrophoresis (2D-DIGE) analysis of NSTEACS patients *vs* healthy controls and STEACS patients *vs* healthy controls, respectively. Statistical analysis revealed significant alterations in the abundance of 47 protein spots (*p* < 0.05, 13 upregulated and 34 downregulated, Table 1) and 24 protein spots (*p* < 0.05, 13 upregulated and 11 downregulated, Table 2), respectively.

**Table 1:** **List of identified proteins in the 2D-DIGE analysis of NSTEACS patients** ***vs*** **healthy controls**

Spot	Protein	Accession number	Mascot score	Ratio P/C	Theoretical MW (Da)	Theoretical PI	Exp MW (Da)	Exp PI	Biological function
1	Inter-alpha-trypsin inhibitor heavy chain H4	ITIH4_HUMAN	143	1.68	103357.43	6.51	65501	4.98	Inflammation
2	Inter-alpha-trypsin inhibitor heavy chain H4	ITIH4_HUMAN	91	1.76	103357.43	6.51	65295	5.01	Inflammation
3	Inter-alpha-trypsin inhibitor heavy chain H4	ITIH4_HUMAN	81	1.51	103357.43	6.51	65226	5.03	Inflammation
4	Inter-alpha-trypsin inhibitor heavy chain H4	ITIH4_HUMAN	169	1.71	103357.43	6.51	65158	5.12	Inflammation
5	Ceruloplasmin	CERU_HUMAN	100	1.52	122205.19	5.44	64849	5.18	Transport
6	Ceruloplasmin	CERU_HUMAN	163	1.56	122205.19	5.44	67216	5.34	Transport
7	Complement factor H	CFAH_HUMAN	102	-1.69	137052.59	6.12	66942	5.45	Immunity
8	Complement factor H	CFAH_HUMAN	171	-1.53	137052.59	6.12	67045	5.47	Immunity
9	Complement factor H	CFAH_HUMAN	242	-1.63	137052.59	6.12	67113	5.49	Immunity
10	Complement C1r subcomponent	C1R_HUMAN	225	1.59	78213.16	5.76	60663	5.38	Inflammation
11	Alpha-1-antichymotrypsin	AACT_HUMAN	104	3.16	47650.87	5.33	55105	4.94	Inflammation
11	Kininogen-1	KNG1_HUMAN	450	3.16	69896.70	6.23	55105	4.94	Inflammation
12	Antithrombin-III	ANT3_PONAB	150	2.86	49039.14	5.95	54556	5.27	Coagulation
13	Inter-alpha-trypsin inhibitor heavy chain H4	ITIH4_HUMAN	165	-1.81	103357.43	6.51	51674	5.50	Inflammation
14	Beta-Ala-His dispeptidase	CNDP1_HUMAN	431	1.53	56692.06	5.14	56477	4.68	Proteolysis
14	Vitronectin	VTNC_HUMAN	221	1.53	54305.59	5.55	56477	4.68	Inflammation
14	Alpha-1-antichymotrypsin	AACT_HUMAN	131	1.53	47650.87	5.33	56477	4.68	Inflammation
15	Gamma fibrinogen	FIBG_HUMAN	182	-2.21	48483.03	5.24	49821	5.07	Coagulation
16	Ceruloplasmin	CERU_HUMAN	173	-2.63	122205.19	5.44	45841	5.09	Transport
16	Apolipoprotein A-IV	APOA4_HUMAN	121	-2.63	45399.06	5.28	45841	5.09	Transport
17	Vitamin D binding protein	VTDB_HUMAN	163	-1.88	52963.65	5.40	47522	5.17	Transport
18	Hemopexin	HEMO_HUMAN	140	-2.37	51676.37	6.55	44297	5.42	Transport
19	Complement factor H related protein-1	FHR1_HUMAN	188	-3.19	35738.20	7.10	44400	5.56	Inflammation
20	Catalytic chain carboxypeptidase N	CBPN_HUMAN	228	-1.58	50034.39	6.88	44743	6.19	Peptidase
21	Leucin rich alpha-2-glicoprotein	A2GL_HUMAN	143	-2.01	34346.41	5.66	45326	4.47	Unknown
22	Kininogen-1	KNG1_HUMAN	79	-1.71	69896.70	6.23	40969	4.53	Inflammation
23	Heparin cofactor 2	HEP2_HUMAN	103	-1.51	54960.02	6.26	36234	4.64	Coagulation
24	Complement C1r subcomponent	C1R_HUMAN	105	-1.75	78213.16	5.76	35445	5.13	Inflammation
25	Apolipoprotein E	APOE_HUMAN	87	-1.80	34236.68	5.52	35960	5.38	Transport
26	Ficolin-3	FCN3_HUMAN	127	-1.83	32902.98	6.22	36612	5.68	Inflammation
27	Complement factor H	CFAH_HUMAN	199	-1.93	137052.59	6.12	36509	5.83	Inflammation
28	Complement factor H	CFAH_HUMAN	169	-1.78	137052.59	6.12	36268	5.99	Inflammation
29	Antithrombin-III	ANT3_HUMAN	138	-2.08	49039.14	5.95	21652	5.18	Coagulation
30	Apolipoprotein A-I	APOA1_HUMAN	101	-2.68	28078.62	5.27	23333	5.48	Transport
31	Retinol binding protein 4	RET4_HUMAN	80	-1.52	21071.60	5.27	17844	5.61	Transport
32	Kininogen-1	KNG1_HUMAN	142	-2.37	69896.70	6.23	13452	4.68-	Inflammation
33	Kininogen-1	KNG1_HUMAN	143	-2.37	69896.70	6.23	11016	4.82	Inflammation
34	Apolipoprotein A-IV	APOA4_HUMAN	204	-2.97	45399.06	5.28	7173	4.74	Transport
35	Fibronectin	FINC_HUMAN	185	1.66	259562.86	5.40	55654	5.98	Inflammation
35	Alpha-2-macroglobulin	A2MG_HUMAN	174	1.66	160809.88	5.98	55654	5.98	Coagulation

PI: isoelectric point; MW: molecular weight; Prot score: score in MASCOT; Ratio P/C: variation between the patient group and the control group.

**Table 2:** **List of identified proteins in the 2D-DIGE analysis of STEACS patients** ***vs*** **healthy controls**

Spot	Protein	Accession number	Mascot score	Ratio P/C	Theoretical MW (Da)	Theoretical PI	Exp MW (Da)	Exp PI	Biological function
1	Inter-alpha-trypsin inhibitor heavy chain H2	ITIH2_HUMAN	143	1.58	72452.28	5.75	105156	4.61	Inflammation
2	Inter-alpha-trypsin inhibitor heavy chain H2	ITIH2_HUMAN	91	1.74	72452.28	5.75	104880	4.62	Inflammation
3	Inter-alpha-trypsin inhibitor heavy chain H2	ITIH2_HUMAN	81	1.76	72452.28	5.75	104605	4.70	Inflammation
4	Inter-alpha-trypsin inhibitor heavy chain H2	ITIH2_HUMAN	127	1.56	72452.28	5.75	104605	4.72	Inflammation
5	Inter-alpha-trypsin inhibitor heavy chain H2	ITIH2_HUMAN	169	1.87	72452.28	5.75	104605	4.64	Inflammation
6	Inter-alpha-trypsin inhibitor heavy chain H2	ITIH2_HUMAN	162	2.01	72452.28	5.75	104743	4.67	Inflammation
7	Inter-alpha-trypsin inhibitor heavy chain H2	ITIH2_HUMAN	114	1.97	72452.28	5.75	104468	4.69	Inflammation
8	Inter-alpha-trypsin inhibitor heavy chain H2	ITIH2_HUMAN	126	1.51	72452.28	5.75	104468	4.74	Inflammation
9	Fibronectin	FINC_HUMAN	181	-1.90	259562.86	5.39	95790	5.35	Inflammation
10	Inter-alpha-trypsin inhibitor heavy chain H1	ITIH1_HUMAN	123	1.53	71415.01	6.36	89463	5.75	Inflammation
11	Haptoglobin	HPT_HUMAN	107	-2.79	43349.01	6.13	44644	5.16	Homeostasis
12	Alpha-1B-glicoprotein	A1BG_HUMAN	218	-3.64	51921.66	5.63	44003	5.30	Unknown
13	Hemopexin	HEMO_HUMAN	467	-8.90	4295.43	6.43	61518	5.92	Transport
14	Kallistatin	KAIN_HUMAN	107	-2.27	46355.24	7.88	61115	5.95	Protease inhibitor
15	Beta-2-glicoprotien 1(ApoH)	APOH_HUMAN	80	-2.80	36254.60	8.37	61035	6.09	Coagulation
16	Peroxiredoxin-2	PRDX2_HUMAN	131	-4.36	21760.73	5.67	18607	5.30	Anti-apoptosis
17	Antithrombin III	ANT3_HUMAN	100	-4.23	49039.14	5.95	14232	5.44	Coagulation
18	Carbonic anhydrase	CAH1_HUMAN	124	-5.02	28739.02	6.63	25844	6.17	Metabolism

PI: isoelectric point; MW: molecular weight; Prot score: score in MASCOT; Ratio P/C: variation between the patient group and the control group.

**Figure 1:**
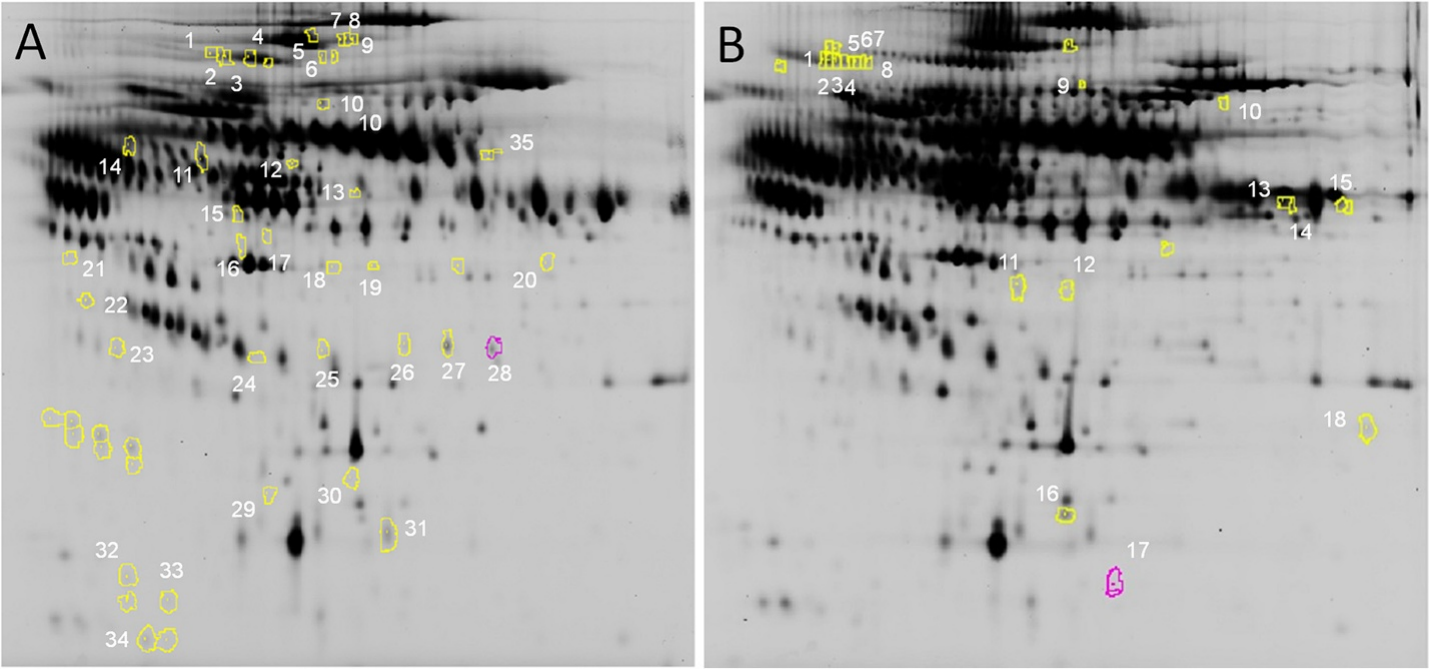
**Differential protein spots obtained in both 2D-DIGE analyses.** **(A)** NSTEACS vs controls and **(B)** STEACS vs controls.

These differentially expressed spots were excised from silver stained gels, digested with trypsin and analyzed by MALDI-TOF/TOF. The ensuing database search identified 23 differentially expressed proteins in NSTEACS patients *vs* healthy controls and 11 in STEACS patients *vs* healthy controls. The spot maps of both analyses are shown in Figure 1 and complete data of identified spots are shown in Tables 1 and 2.

**Figure 2:**
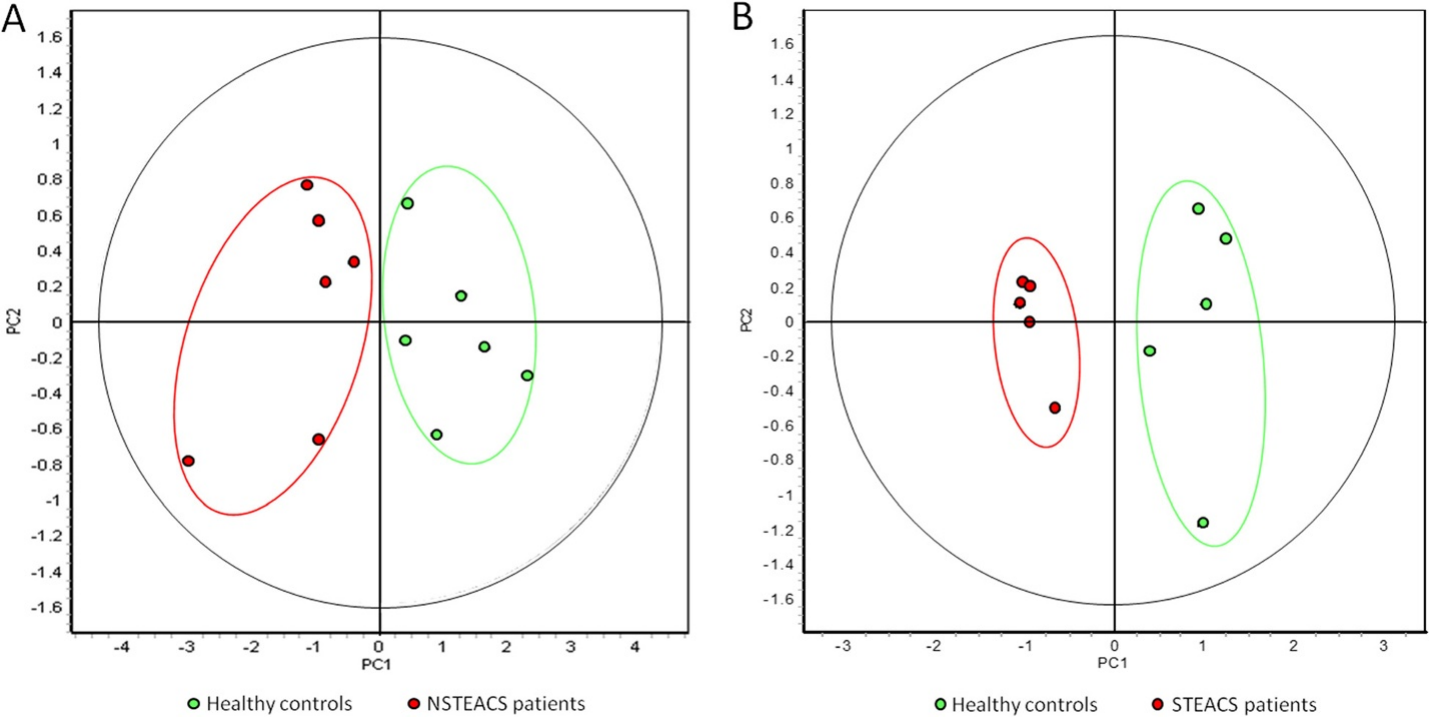
**Score plots obtained in the PCA for data from the NSTEACS vs controls (A) and STEACS vs controls (B) 2D-DIGE analyses.**

Principal component analysis (PCA) was used to reduce the complexity of the multidimensional dataset, providing a clearer overview to better reveal trends within the data. In both 2D-DIGE analyses, this analysis efficiently discriminated NSTEACS (Figure 2A) or STEACS patients (Figure 2B) from healthy controls plasma samples with perfect separation of samples by the first principal component (PC1). In this plot, healthy controls plasma samples were scattered on the left side of the plot and NSTEACS or STEACS plasma samples were located on the right.

**Figure 3:**
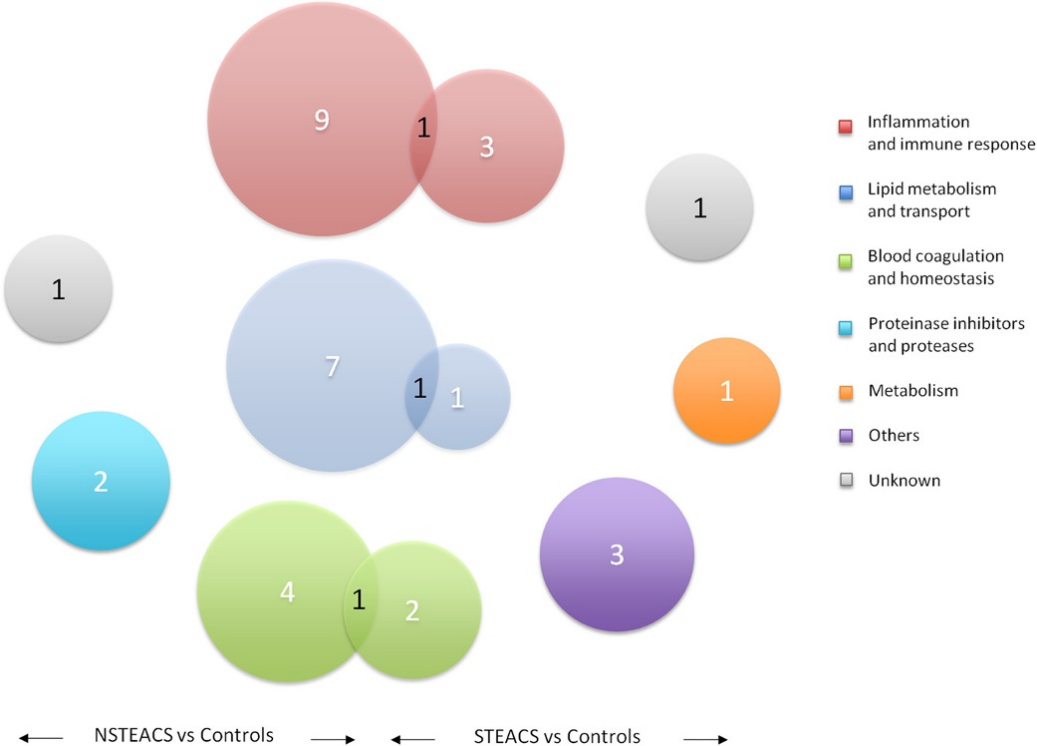
**Venn diagram showing the proteins identified in both 2D-DIGE analyses.**

The majority of proteins identified in both 2D-DIGE analyses could be classified into seven functional categories (Figure 3), namely: *Metabolism* (~3.2%), *Lipid Metabolism and Transport* (~29.3%), *Inflammation and Immune response* (~25.8%), *Blood homeostasis and coagulation* (~16.2%); *Proteases and protease inhibitors* (~9.7%), *Others* (~6.4%) and Unknown (~6.4%). These functional categories were mainly determined using the Uniprot database:

1. *Metabolism:* Carbonic anhydrase.
2. *Lipid metabolism and transport*: ceruloplasmin, apolipoprotein A-IV, vitamin D binding protein, apolipoprotein E, apolipoprotein AI, retinol binding protein 4 and hemopexin.
3. *Inflammation and immune response:* vitronectin, ficolin 3, inter-alpha-trypsin inhibitor heavy chain H1, inter-alpha-trypsin inhibitor heavy chain H2, inter- alpha-trypsin inhibitor heavy chain H4, complement C1r, alpha-1-antichymotrypsin, kininogen-1, complement factor H, complement factor H related protein-1 and fibronectin.
4. *Blood coagulation and homeostasis:* antithrombin III, gamma fibrinogen, heparin cofactor 2, alpha-2-macrogobulin and beta-2-glicoprotein 1.
5. *Proteinase inhibitors and proteases:* beta-Ala-His dipeptidase, carboxypeptidase N catalytic chain and kallistatin.
6. *Others:* haptoglobin and peroxiredoxin 2.
7. *Unknown:* alpha-1B-glicoprotein and leucine-rich alpha-2-glycoprotein.

### Annotation and functional enrichment

Functional analysis of the altered proteins was performed using DAVID v6.7. Molecular functions and biological processes were explored through the functional annotation tool to generate clusters of overrepresented Gene Ontology (GO) terms. Molecular function analysis in NSTEACS showed that a big number of the altered proteins had enzyme inhibitor activity. High fold enrichment was also related to immune response and inflammation. Complement activation, homeostasis and coagulation together with immune response and inflammation are the biological processes more representative in which altered proteins are primarily involved.

Pathway analysis revealed a significant enrichment in Intrinsic Prothrombin Activation Pathways (BIOCARTA) as well as in complement and coagulation cascades (KEGG). In the case of STEACS, pathways analysis did not reveal a significant enrichment.

Functional annotation clustering tool was used to gather molecular function and biological process results. Seven significant clusters were obtained: inflammation and immune response (enrichment score: 5.62), enzyme inhibitor activity (enrichment score: 4.66), metabolism (2.96), response (2.46), regulation (2.36) binding-related (enrichment score: 2′3), and homeostasis (enrichment score: 2.19).

### Protein-protein interaction

**Figure 4:**
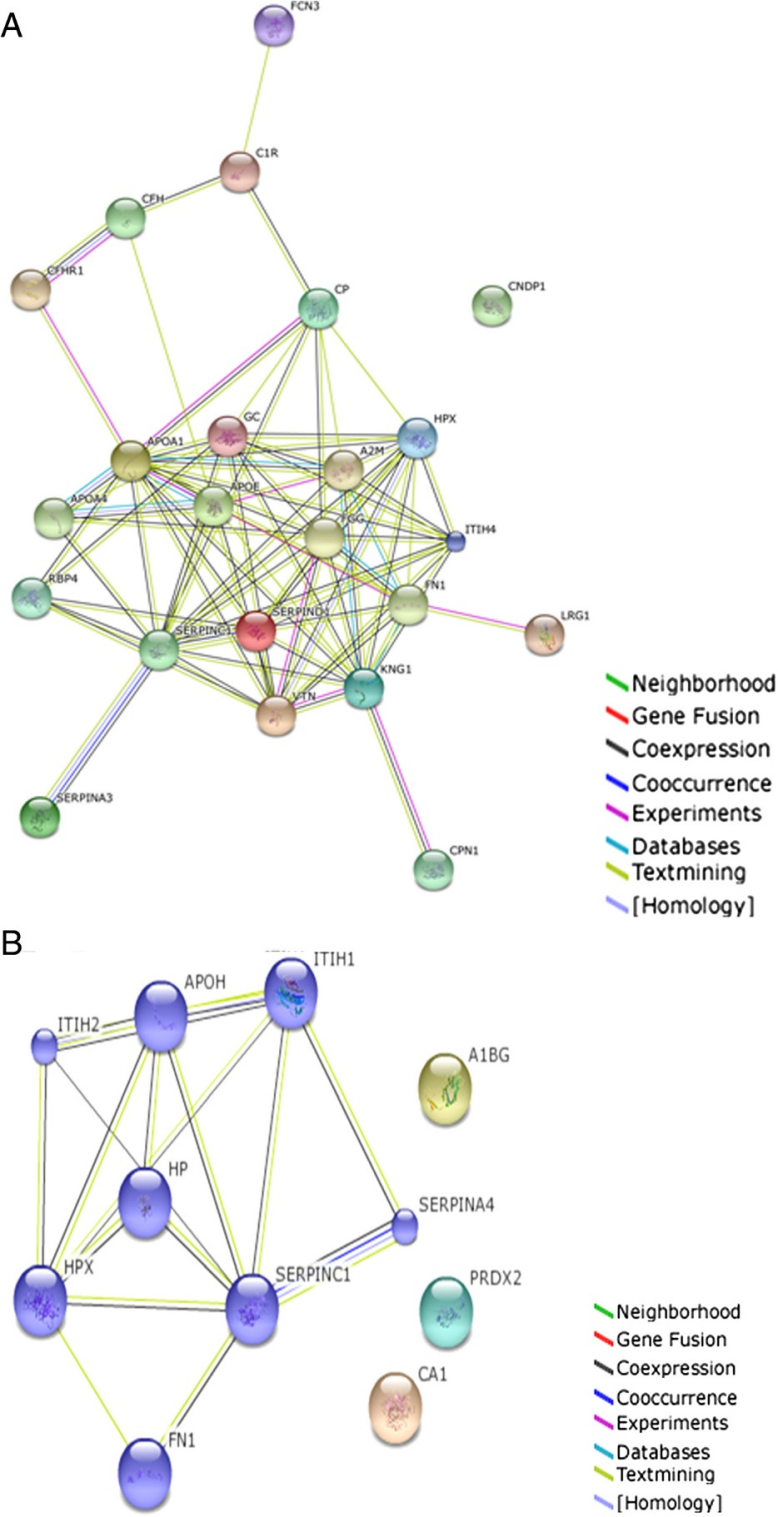
**Protein-protein interaction networks were studied using the STRING v9.1 web-tool.** **(A)** NSTEACS Protein-Protein interaction networks. **(B)** STEACS protein-Protein interaction networks. Different line colours represent the types of evidence for the association.

The 23 dysregulated proteins determined in the NSTEACS 2D-DIGE experiment were introduced into the web-tool STRING v9.1 to generate protein-protein interactions networks. After clustering, seven functional modules forming tightly connected clusters can be observed (Figure 4A). Regarding STEACS 2D-DIGE experiment, after clustering two functional clusters were observed (Figure 4B).

### Validation by Western blot

**Figure 5:**
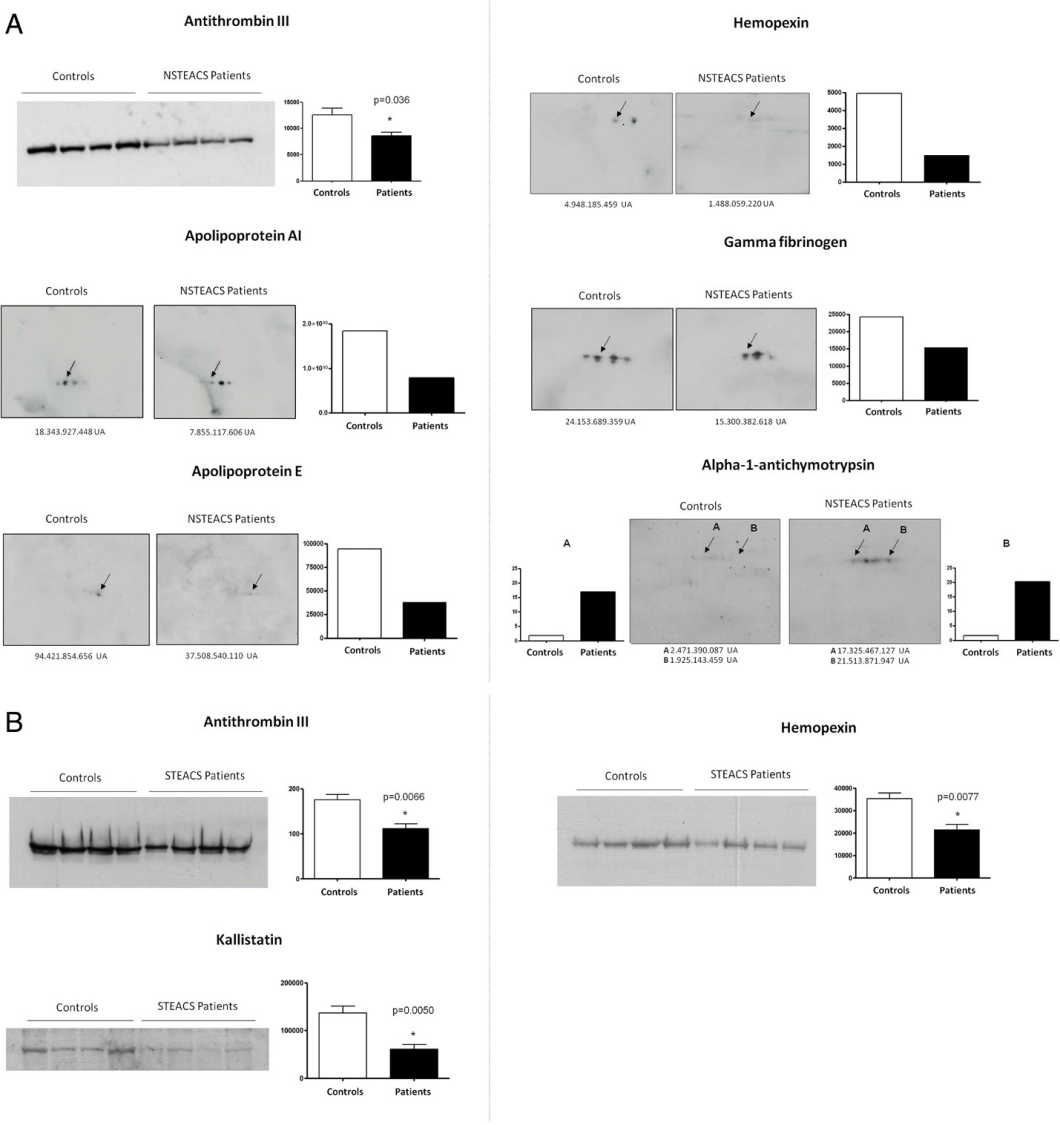
**Western blot validation.** **(A)** NSTEACS vs controls and **(B)** STEACS vs controls.

To confirm the proteomic results, western blot was performed to antithrombin III (~49 kDa), alpha-1-antiquimiotripsin (~48 kDa), hemopexin (~50 kDa), apolipoprotein AI (~30 kDa), gamma fibrinogen (~48 kDa), apolipoprotein E (~36 kDa), and kallistatin (~46 kDa) using a densitometry software (Quantity One, BioRad). In case of hemopexin and antithrombin III, these proteins were validated in both, NSTEACS and STEACS patients (Figure 5).

### NSTEACS patients vs healthy controls

For antithrombin III, the analysis of band intensity showed a statistically significant decreasing expression in NSTEACS patients compared with healthy controls (p = 0.036). In the 2D western blot of alpha-1-antichymotrypsin several protein isoforms were observed. The two isoforms that were found differentially expressed in the 2D-DIGE were also increased.

The 2D western blot of apolipoprotein E and hemopexin confirmed the results obtained in the 2D-DIGE (−1.80 and −2.37, respectively) showing a decreasing expression in NSTEACS patients. Gamma fibrinogen showed several isoforms. The differentially reduced isoform found in the 2D-DIGE analysis (−1.58) also showed diminished levels of expression in the patient group NSTEACS. Similar results were obtained with apolipoprotein AI, where 2D western blot confirmed the decreased expression levels in patients with NSTEACS previously observed (−2.68).

### STEACS patients vs healthy controls

The analysis of band intensity for antithrombin III, hemopexin and kallistatin showed, in all three cases, a statistically significant decrease in their expression levels in patients with STEACS versus healthy controls with significance values p = 0.0066, p = 0.0077 and p = 0.005 respectively, confirming the results obtained in 2D-DIGE.

### Validation by Selected Reaction Monitoring (SRM)

Additionally, in order to complete the validation of the results obtained in the two previous proteomic analyses, several proteins with altered expression profiles detected in the different 2D-DIGE analyses were monitored by SRM.

### NSTEACS patients vs healthy controls

**Figure 6:**
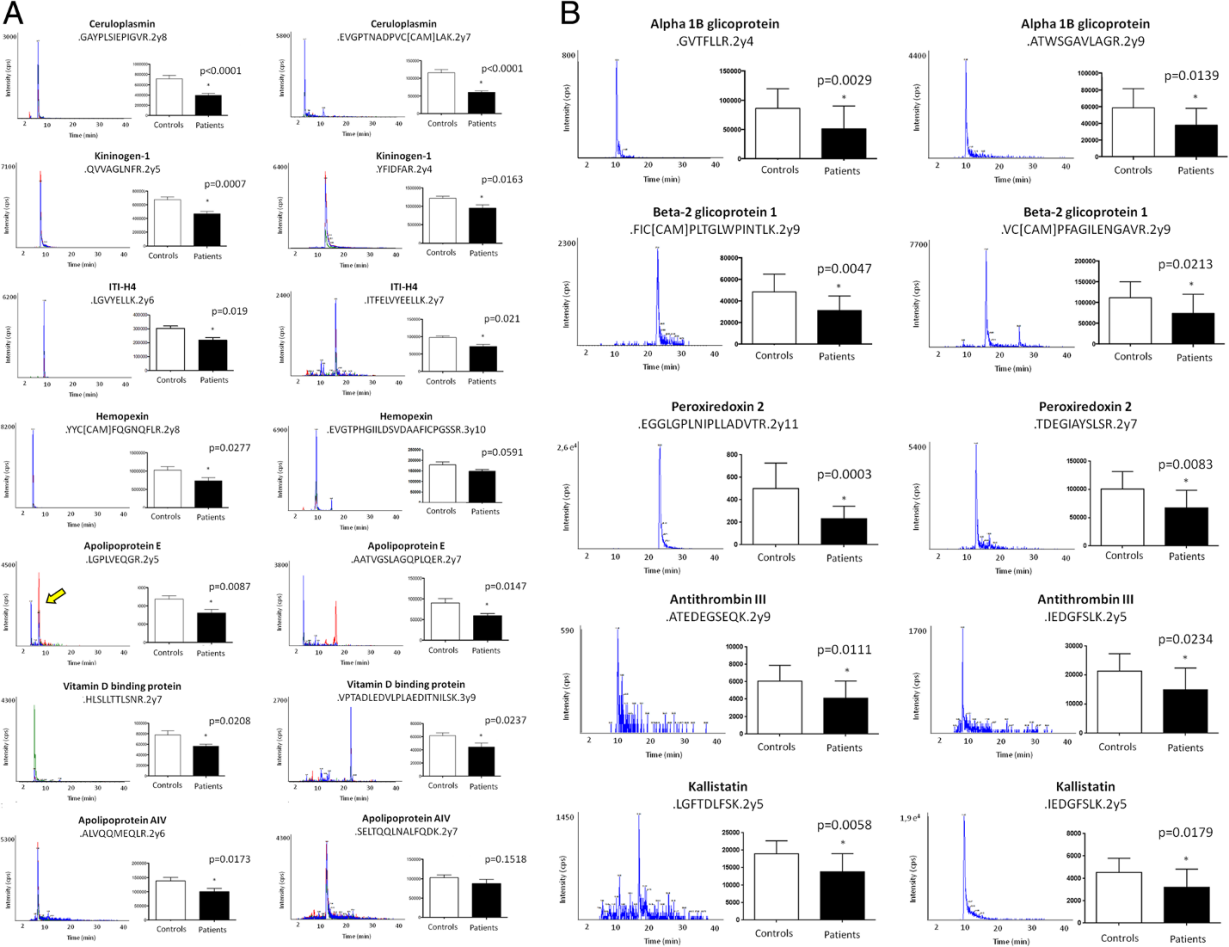
**SRM validation.** **(A)** NSTEACS vs controls and **(B)** STEACS vs controls. The yellow narrow shows the SRM transition of interest.

The proteins ceruloplasmin (p < 0.001 and p < 0.001), inter alpha trypsin inhibitor heavy chain H4 (p = 0.019 and p = 0.021), apolipoprotein E (p = 0.0087 and p = 0.0147), vitamin D binding protein (p = 0.0208 and p = 0.0237) and kininogen-1 (p = 0.0007 and p = 0.0163) showed statistically significant differences in both peptides after statistical analysis using a Student *t*-test (p < 0.05) (Figure 6A).

Hemopexin (p = 0.0277 and p = 0.0591) and apolipoprotein A-IV (p = 0.0173 and p = 0.1518) showed significant differences in one of the two monitored peptides. In the case of hemopexin, one of the two peptides was very close to statistical significance (p = 0.0591) and since it had been previously validated by western blot this validation by SRM was accepted.

### STEACS patients vs healthy controls

Similarly, an independent group of samples (n = 15 STEACS patients, n = 15 healthy controls) was used. Alpha 1B-glycoprotein (p = 0.0029 and p = 0.0139), beta-2 glycoprotein-1 (apo H) (p = 0.0047 and p = 0.0213), peroxiredoxin 2 (p = 0.0003 and p = 0.0083), antithrombin III (p = 0.0111 and p = 0.0234) and kallistatin (p = 0.0058 and p = 0.0179) showed statistically significant differences in both peptides after statistical analysis using Student’s *t*-test (Figure 6B). Results obtained for antithrombin III and kallistatin were validated by western blot and SRM.

Globally, the results obtained by these two different validation methods confirmed those obtained from both plasma proteomic analyses using the DIA module.

## Discussion

Because of the current clinical tests for ACS just permits its diagnosis after the onset of the syndrome, the discovery of new biomarkers reflecting the development of the atherosclerotic process results critical.

For studying potential candidates, it is previously necessary to consider the basic characteristic that every clinical biomarker should meet; high sensitivity and high specificity. Sensitivity, in many cases, depends on the technique used and it usually improves with different generations of reagents as it has happened with troponin and ultrasensitive reactive C protein. Specificity, however, is a parameter that must be carefully considered from the outset. In our study, we found a total number of 31 altered proteins. Despite this apparently attractive result, many of these proteins do not offer the previously mentioned characteristic of an ideal biomarker. This happens with vitamin D-binding protein (VDBP) and the family of inter-alpha trypsin inhibitors which have been related to different inflammatory diseases such as rheumatoid arthritis, liver cirrhosis and Crohn’s disease [12, 13]. Since they have been also found altered in a number of pathological states, their lack of specificity prevents its clinical use for diagnosis of ACS.

### Interesting proteins with potential clinical use

Fortunately, many of identified proteins are interesting in terms of new biomarkers discovery or better understanding of physiopathology of ACS. In this sense, one of the most remarkable results was the alteration of three proteins with antioxidant properties; haptoglobin, 1B-glycoprotein and peroxiredoxin-2 [14, 15]. In all cases, decreased plasma levels were found in STEACS patients. Therefore, we consider that this alteration could trigger the damage caused by reactive oxygen species (ROS) due to the lack of protective antioxidant effect [16–18]. Similarly, decreased levels of ceruloplasmin and hemopexin could produce an increase of free oxygen promoting tissue oxidation during the atherogenic process due to its ability to catalyse the reduction of oxygen molecules [19] and protect against oxidation of the LDL caused by hemoproteins, respectively [16, 17]. In this group of proteins whose decreased plasma levels may predispose to suffer ACS, we also include kallistatin, a member of the superfamily of serine protease inhibitors with various effects including powerful vasodilator activity, inhibition of angiogenesis and anti-inflammatory activity [20–22].

Extensively known is the role of apolipoproteins in the metabolism and transport of cholesterol. We found several apolipoproteins; ApoA-I, ApoA-IV, ApoE and ApoH with decreased plasma levels in ACS patients. Due to their biological functions, their alterations could favour the appearance of a pro-thrombotic state and the progression of atherosclerosis [23–27].

Coagulation is a basic phase in ACS onset. Fibrinogen and antithrombin III are currently used in clinical practice for the diagnosis of different coagulation alterations. In this group of proteins we also include kininogen-1, a multifunctional protein that participates in the intrinsic pathway of the coagulation as a part of the kallikrein-kinin system [28, 29] inhibiting platelet aggregation induced by thrombin and plasmin [30, 31]. We hypothesize that decreased plasma levels of kininogen-1 could promote platelet aggregation and trigger the progression of atherosclerosis.

One of the most exciting results that we observed in the STEACS plasma proteome was the alteration of carbonic anhydrase. This protein catalyses the rapid conversion of carbon dioxide and water to bicarbonate and free protons to maintain the acid–base balance in the blood and other tissues. It decreased levels in STEACS patients permits us to postulate that tissue hypoxia carries a cardiac muscle intracellular acidosis situation caused by the production of lactic acid derived from anaerobic glycolysis. This state of acidosis would modify the reaction equilibrium in the opposite direction catalysed by the enzyme to prevent the formation and release of higher acid content in the cells. Since carbonic anhydrase was altered in STEACS but not in NSTEACS patients, its decreased plasma levels may have potential to differentiate the extent of cardiac necrosis in ACS.

In summary, ACS is a multifactorial disease in which several processes contribute to the development of the plaque. For this reason, we considered that there is not the possibility of defining a single diagnostic biomarker. In contrast, a panel of biomarkers reflecting these processes could help clinicians to correctly and individually diagnose the risk of every patient of suffering an ACS. In our opinion, this panel should be formed by proteins related to oxidative damage (i.e. hemopexin and ceruloplasmin), coagulation (i.e. kininogen-1 and antithrombin III) and hypoxia (carbonic anhydrase), which were all of them found altered in this study.

### Future lines of research

The results obtained in this study also permits to picture some interesting lines of research for the future. The relationship between different cardiovascular diseases such as ACS and aortic stenosis (AS) could be elucidated through a deeper knowledge of the plasma proteome. In this sense, we obtained statistical differences in two proteins; alpha-1-antichymotrypsin and leucine-rich alpha-2-glycoprotein that we had previously found altered in AS [32]. We believe that these two proteins could play a role in the development of both inflammatory pathologies and permit us to suggest new lines of research to study the possible relationship between AS and atherosclerosis and the differences between these two processes.

## Conclusions

In the present study, our results from both 2D-DIGE analyses permitted us to suggest that NSTEACS patients had a characteristic plasma proteomic profile that differentiates them from those patients who have suffered a STEACS. Although in both 2D-DIGE experiments some patients had previously suffered ACS, as it was explained in detail in the Material and method section any previous disease or surgery should have occurred in a six months period before being included in the study. In our opinion, this period of time is broad enough to consider that the existence of a previous ACS in any patient would have no influence on the protein pattern found in our studies.

Additionally, in spite of the large variety of biological processes of the identified proteins, based on our previous experience in secretome studies, the tissue is the origin of many of them [33, 34]. As previously explained in the Background section, the wide number of biochemical disorders secondary to hypoxia suffered by the myocardium lead to tissue breakdown and the releasing of a large number of proteins of potential interest into the blood stream. In fact, among the proteins forming the characteristic plasma proteomic profile for STEACS patients appeared decreased expression levels of three proteins with antioxidant activity (1B-glycoprotein, peroxiredoxin-2 and haptoglobin) which will possible catalyze the damage caused by the plasma ROS in the endothelium. In spite of a direct NSTEACS vs STEACS 2D-DIGE would probably confirm these results, we are strongly confident that the global proteome described in this study permit us to suggest the need to consider the use of a panel of biomarkers as the most useful tool for early diagnosis of ACS.

## Methods

### Patient selection and sample collection

The study was conducted according to the Declaration of Helsinki and it was approved by the ethics committee of the Hospital Virgen de la Salud (Toledo). All the patients and controls signed an informed consent before their inclusion in the study.

Three experimental groups were selected: 1) NSTEACS patients, 2) STEACS patients and 3) healthy controls. Blood samples (K3 EDTA) of NSTEACS and STEACS patients and sex and age matched healthy subjects were recruited from the Cardiology Service of Hospital Virgen de la Salud (Toledo, Spain). In case of ACS patients, the samples were obtained in the onset of the syndrome (t = 0 h) defined as the moment when the coronary event is diagnosed.

Different pathologies and situations were considered as exclusion criteria. Tumour inflammatory diseases, alterations of coagulation, the existence of significant heart disease unrelated to the disease or its risk factors (valvular, pericardial affectations or cardiomyopathies), chronic treatments, except for ischemic heart disease and its risk factors, coronary angiograms normal ejection fractions less than 0.45, major trauma, thromboembolic events, revascularization or having undergone surgery within six months before the start of the study. The control group included 30 healthy volunteers with normal coronary arteries and up to two cardiovascular risk factors who attended the Department of Hemodynamic for various reasons. All healthy volunteers and patients had a sex and age distribution with no significant differences between them (Table 3).

**Table 3:** **Baseline characteristics of healthy controls and ACS patients included in the study**

	Healthy controls (n = 30)	NSTEACS patients (n = 30)	STEACS patients (n = 30)
**Personal data**			
Age (years) ± SD	64.2 ± 11,5	68.3 ± 9.8	67.1 ± 8.7
Sex (Male/Female)	17/18	27/8	21/6
**Risk factors**			
Smokers	3	10	9
Ex-smokers	3	8	7
Diabetes mellitus	1	5	7
Dyslipidemia	6	23	17
Hypertension	14	22	22
Renal disease	0	3	3
**Biochemical data**			
Total cholesterol (mg/	182 ± 37	165 ± 27	172 ± 28
LDL (mg/dL)	103 ± 36	99 ± 28	109 ± 31
HDL (mg/dL)	50 ± 12	49 ± 8	47 ± 9
Triglycerides (mg/dL)	123 ± 50	144 ± 59	161 ± 43
**Background data**			
Previous ACS	0%	8	4
Statins	0%	17	10

### Depletion of high-abundant plasma proteins

In order to increase the concentration of low-abundant proteins in plasma samples, the 14 most abundant plasma proteins were specifically removed by affinity chromatography using the Multiple Affinity Removal Column (MARS Hu-14, Agilent Technologies). This technique has proven to be an interesting tool in the search for plasma biomarkers [32, 35, 36].

Depletion was performed by high performance liquid chromatography (HPLC) using a 1200 series HPLC chromatograph (Agilent Technologies) equipped with a manual injector (9725i, Agilent Technologies). After several chromatographic cycles, aliquots of the flow-through fractions containing low-abundance proteins were combined and desalted using centrifugal filter devices with a 3 kDa cut off (Amicon Ultra, Millipore). These samples were stored at -80°C prior to analysis and the protein concentration was determined using the Bradford-Lowry method [37].

### Two-dimensional differential in gel electrophoresis (2D-DIGE)

NSTEACS plasma samples (n = 5) and healthy control samples (n = 5) or STEACS plasma samples (n = 6) and healthy control samples (n = 6) with similar clinical parameters were used in both 2D-DIGE analyses. This technique is based on the specific linkage of three fluorochromes with differential spectral properties (Cy2, Cy3 and Cy5). It permits the separation of multiple samples in a single gel increasing the reproducibility and reliability of the analysis of differential expressed proteins.

Proteins were labelled according to the manufacturer’s instructions (GE Healthcare), such that 50 μg of depleted plasma proteins from NSTEACS or STEACS patients and healthy controls were labeled with 400 pmol of N-hydroxysuccinimide Cy3 or Cy5 fluorescent cyanine dye and the labeling reaction was then quenched with 0.2 mM lysine. All experiments included an internal standard containing equal amounts of each protein extract labeled with 400 pmol of N-hydroxysuccinimide Cy2 dye. The internal standard and two additional plasma samples (NSTEACS/STEACS and control) were combined and run on a single gel (150 μg of total protein). Protein extracts were diluted in rehydration buffer (30 mM TRIS, 7 M Urea, 2 M Thiourea, 4% CHAPS) and applied to 24 cm pH 4–7 IPG strips. The first dimension was run on the IPGphor IEF II System (GE Healthcare) in darkness, as follows: 500 V for 30 minutes, a linear gradient to 3500 V over 3 h, 3500 V for 3 h, a linear gradient to 6000 V over 3 h, and 6000 V until 69000 v/h. After the first dimension, the strips were equilibrated in SDS-equilibration buffer (1.5 M Tris/HCl [pH 8.8], 6 M Urea, 87% Glycerol and 2% SDS).

To increase the reproducibility an electrophoresis system “Ettan DALTsix” (GE Healthcare) was used for the polymerization of the gels. To run the second dimension 10% acrylamide gels were used. To do this, 1.5 M Tris–HCl pH 8.8 was used, 10% SDS (w/v) and milliQ water were used to obtain the desired concentration of acrylamide. APS and TEMED (BioRad) were also added to catalyst the polymerization reaction. After preparing the gels, each IPG strip was placed horizontally on the top of the gel, avoiding bubbles to remain trapped between the strip and the surface of the gel. Electrophoresis took place at 25°C, firstly applying 5 W/gel for 20 min and then 1 W/gel overnight in the presence of running buffer (25 mM Tris, 0.2 M glycine, 3 mM SDS).

### Image acquisition and analysis

After SDS-PAGE, the gels were scanned on a Typhoon TRIO fluorescence gel scanner (GE Healthcare) using appropriate individual excitation and emission wavelengths, filters and photomultiplier values sensitive for each of the Cy3, Cy5 and Cy2 dyes.

Relative protein quantification was performed on NSTEACS and STEACS patients and healthy control samples using DeCyder software v6.5 (GE Healthcare) and the multivariate statistical Extended Data Analysis (EDA) module. The DIA module simultaneously detected the 3 images of each gel (the internal standard plus two samples), measured the spot abundance in each image, and expressed these values as Cy3/Cy2 and Cy5/Cy2 ratios. These DIA datasets were then analysed using the Biological Variation Analysis (BVA) module, which matched the spot maps and compared the Cy3/Cy2 and Cy5/Cy2 ratios. Differences in protein abundance >1.5-fold were considered significant. Statistical analysis using Student’s *t*-test was performed to detect changes in protein expression, with p ≤ 0.05 accepted as significant.

Finally, a multivariate analysis was performed through a Principal Component Analysis (PCA) using the algorithm included in the EDA module of DeCyder software based on the spots that matched across all gels.

### Conventional two-dimensional electrophoresis (2-DE)

For the protein spots identification 2-DE gels were used. Plasma samples were diluted in rehydration buffer and applied to pH 4–7 IPG strips, which were subsequently equilibrated as previously described [32, 38, 39]. For the second-dimension separation, 10% SDS-PAGE was performed according to Laemmli [40] using a Protean II system (BioRad) at 4°C and 25 mA/gel. The gels were fixed overnight and Silver Stained (GE Healthcare) following the manufacturer’s instructions and finally scanned with a GS-800 Calibrated Densitometer (BioRad).

### Protein identification by MALDI-TOF/TOF and database searching

Protein spots from 2-DE gels were excised manually, automatically digested with an Ettan Digester (GE Healthcare) and identified at the Proteomic Unit of Hospital Nacional de Paraplejicos. In-gel trypsin digestion was performed according to Schevchenko *et al*. [41] with minor modifications. Peptides were extracted with 60% acetonitrile (ACN) in 0.1% formic acid (99.5% purity; Sigma Aldrich). Samples were dried in a speed vac and re-suspended in 98% water with 0.1% formic acid (FA) and 2% ACN. An aliquot of each digestion was mixed with an aliquot of the matrix solution (3 mg/mL matrix α-cyano-4-Hydroxycinnamic acid: CHCA, Sigma Aldrich) in 30% ACN, 15% 2-propanol and 0.1% TFA, and this was pipetted directly onto the stainless steel sample plate of a 384 Opti-TOF 123 × 81 mm MALDI mass spectrometer (ABsciex) and dried at room temperature. MALDI-MS/MS data were obtained using an automated analysis loop in a 4800 Plus MALDI TOF/TOF Analyzer (ABsciex).

MALDI-MS and MS/MS data were combined using GPS Explorer Software Version 3.6 to search a non-redundant protein database (Swissprot 56.5) with the Mascot software version 2.2 (Matrix Science) applying the following settings: 50 ppm precursor tolerance, 0.6 Da MS/MS fragment tolerance, 1 missed cleavage allowed, carbamidomethylcysteines (variable) and methionine oxidation (fixed) as modifications. The MALDI-MS/MS spectra and database search results were manually inspected in detail using the previous software.

### Pathways analysis

Functional classification and protein interactions were investigated using publicy available bioinformatics tools: Database for Annotation, Visualisation and Integrated Discovery (DAVID v6.7) and Search Tool for the Retrieval of Interacting Genes/Proteins (STRING v9.1). DAVID bioinformatics provides an integrated biological knowledgebase and analytic tools aimed at systematically extracting biological meaning from large gene/protein lists derived from high-throughput experiments. STRING is a database and web resource dedicated to protein–protein interactions, including both physical and functional interactions, that maps all interaction evidence onto a common set of genomes and proteins. Proteins with different expression between IS patients and control were uploaded to DAVID and STRING for functional and protein interaction analyses, respectively.

### Western blot

For the validation process, independent groups of samples from healthy controls and NSTEACS or STEACS patients were used. All samples were depleted and quantitated using the Bradford-Lowry method [37]. One-dimensional (1D) and two-dimensional (2D) western blots were used.

20 ug of protein from every depleted plasma sample were mixed with loading buffer (10% SDS, 87% glycerol, 2-mercaptoethanol 5% Bromophenol blue and 0.002% Tris -HCl 0.5 M pH 6.8) and heated at 95°C for 5 min before application on the gel wells.

Then, electrophoresis was performed using SDS-PAGE different percentages of acrylamide/bisacrylamide (between 8 and 12% acrylamide), depending on the expected molecular mass of the protein to be analyzed. Protein electrophoresis in denaturing conditions was performed as described by Laemmli protocol [40] and immobilization was carried out onto nitrocellulose membranes (BioRad) by semi-dry transfer (BioRad) for 30 min at 20 V, using transfer buffer (25 mM Tris, 151.8 mM glycine and 20% methanol v/v). Then, the nitrocellulose membranes were stained by immersing them in a solution of Ponceau Red S (Sigma) in 0.2% trichloroacetic acid 30% (w/v) sulfosalicylic acid and 30% (w/v) for 1 min, washed with distilled water to remove excess dye and a scanned with the densitometer (GS-800 Calibrated Densitometer, BioRad). To check the performance of the transfer gels were stained with Coomassie blue. Once the proteins immobilized on nitrocellulose membranes, these were blocked in a solution of skimmed milk powder 7.5% (w/v) in PBS/ 0.1% Tween20 overnight at 4°C under stirring. After blocking, all subsequent steps were performed at room temperature and under stirring. The membranes washed three times for 10 min with washing buffer PBS/ 0.1% Tween20. The membranes were incubated for 1 hour with the specific primary antibody diluted in PBS/0.1% Tween20 (Table 4). After making three washes of 10 min in wash buffer, the membranes were incubated for 45 min with the appropriate secondary antibody diluted in PBS/ 0.1% Tween20. Finally, repeated three washes of 10 min and revealed using the method based chemiluminescent oxidation of luminol by the action of hydrogen peroxide catalyzed by horseradish peroxidase (ECL Western Blotting Detection Reagents, GE Healthcare). Computer-assisted densitometric image analysis of digital subtraction images was performed to quantify the proteins. Student’s *t*-test (p < 0.05) was used for the statistical analysis of data.

**Table 4:** **Resume of primary and secondary antibodies used for western blot validation along with dilutions and incubation times**

Protein	Primary antibody	Dilution	Incubation	Secondary antibody	Dilution	Incubation
Gamma fibrinogen	Abcam	1:20000	1 hour	**-**	**-**	**-**
Hemopexin (NSTEACS)	Abcam	1:2000	1 hour	Rabbit True blood eBioscience	1:1000	45 min
Apolipoprotein E	Abcam	1:500	1 hour	IgG antigoat donkey HRP Abcam	1:2000	45 min
Antithrombin III (NSTEACS)	Abcam	1:2000	1 hour	Mouse True blood eBioscience	1:1000	45 min
Alpha-1 antichymotrypsin	Abcam	1:2000	1 hour	Mouse True blood eBioscience	1:1000	45 min
Apolipoprotein AI	Polyclonal	1:50000	1 hour	Rabbit True blood eBioscience	1:10000	45 min
Coagulation Factor XII	Abcam	1:1000	1 hour	Rabbit True blood eBioscience	1:10000	45 min
Antithrombin III (STEACS)	Abcam	1:2000	1 hour	Mouse True blood eBioscience	1:1000	45 min
Hemopexin (STEACS)	Abcam	1:200	1 hour	Rabbit True blood eBioscience	1:10000	45 min
Kallistatin	Abcam	1:2000	1 hour	Rabbit True blood eBioscience	1:10000	45 min

### Selected reaction monitoring

Crude plasma samples were reduced, digested and cleaned with Pep-Clean spin columns (Pierce) according to the manufacturer’s instructions prior to mass spectrometry (MS) analysis in the LC-MS/MS system. It consisted of a TEMPO nano LC system (Applied Biosystems) combined with a nano LC Autosampler coupled to a modified triple quadrupole (4000 QTRAP LC/MS/MS, Applied Biosystems). Theoretical SRM transitions were designed using MRMpilot software v1.1 (ABSciex). An independent group of 15 controls and 15 NSTEACS or STEACS patients were analyzed in the 4000QTrap using a MIDAS acquisition method that included the theoretical transitions.

### Statistical analysis

A Kolmogorov-Smirnov test demonstrated the normal distribution of the population analyzed. A Levene test for homogeneity of variance was performed and the Student *t*-test was used to compare band intensities. SRM statistical analysis was carried out using the GraphPad Prism 4.0 software package and for all tests statistical significance was accepted when p < 0.05.

## Abbreviations

2D-DIGE: Two-dimensional difference in gel electrophoresis
2DE: Two-dimensional electrophoresis
ACN: Acetonitrile
ACS: Acute coronary syndrome
AHA: American heart association
ApoA-I: Apolipoprotein A-I
ApoA-IV: Apolipoprotein A-IV
ApoE: Apolipoprotein E
ApoH: Apolipoprotein H
APS: Ammonium Persulfate
AS: Aortic stenosis
BVA: Biological variation analysis
CK: Creatine kinase
CKMB: Heart type creatine kinase isoenzyme
DIA: Differential in-gel analysis
EDA: Extended data analysis
ESC: European society of cardiology
HPLC: High performance liquid chromatography
MALDI: Matrix-assisted laser desorption/ionization
NSTEACS: non ST-elevation acute coronary syndrome
PCA: Principal component analysis
ROS: Reactive oxygen species
SRM: Selected reaction monitoring
STEACS: ST-elevation acute coronary syndrome
TEMED: Tetramethylethylenediamine
TOF: Time of flight
VDBP: Vitamin D-binding protein.
